# Misdiagnosed Tooth Aspiration in a Young Handicapped Boy: Case Report and Recommendations

**DOI:** 10.1155/2019/8495739

**Published:** 2019-10-23

**Authors:** Thibault Canceill, Rémi Esclassan, Mathieu Marty, Marie-Cécile Valera, Estelle Trzaskawka-Moulis, Emmanuelle Noirrit-Esclassan

**Affiliations:** ^1^Paul Sabatier University, Dental Faculty, Toulouse University Hospital (CHU de Toulouse), 3 Chemin des Maraîchers, 31062 Toulouse Cedex 9, France; ^2^CIRIMAT, University of Toulouse, CNRS, INPT, Université Paul Sabatier, Faculté de Pharmacie, 35 Chemin des Maraichers, 31062 Toulouse Cedex 9, France; ^3^Laboratoire AMIS, UMR 5288 CNRS, 37 Allées Jules Guesde, 31000 Toulouse Cedex 09, France; ^4^INSERM U1048, I2MC, CHU Rangueil, BP 84225, 31432 Toulouse Cedex 4, France; ^5^Dental Faculty, Montpellier University Hospital, 549 av du Professeur Louis Viala, 34295 Montpellier Cedex 5, France

## Abstract

Tooth inhalation remains a rare incident but it may occur during dental care, especially in children. We report here the case of a four-year-old boy with Down syndrome who came to the hospital after a dental trauma. During the extraction procedure, he aspired his maxillary incisor without presenting any signs of respiratory distress and was discharged by the surgical team, who thought that he had swallowed the tooth. Three weeks later, he was admitted to the emergency service because of a pulmonary infection. Two endoscopy interventions under general anesthesia were necessary to recover the foreign body inside the left lung. Because of the multiple symptoms associated with the trisomy 21 syndrome (general hypotonia, impaired immunity, etc.), practitioners should be very mindful of aspiration risks and complications during dental care. The systematic prescription of lung radiography would prevent the onset of pulmonary infections and enable an earlier intervention.

## 1. Introduction

A foreign body may be accidentally aspirated into the airways. This may occur in adults [1, 2] but is more frequent in children [3, 4], especially in boys less than 3 years of age, [5] for whom choking is the leading cause of death [6]. The most commonly aspirated foreign bodies are of organic origin (seeds, nuts, berries, and grains) [7], but inhalation of dental instruments and natural tooth fragments can also happen [8, 9]. In the general population, objects used during dental procedures have been reported as the second most common type of foreign bodies that may be aspired into the lungs [10]. The event can potentially occur during routine clinical procedures with the aspiration into the respiratory tracts of pieces of teeth, crowns, tips of dental instruments, hemostatic dressings, osseous fixation wires, impression materials, and implant instruments [11, 12]. All dental specialties are concerned: prosthodontics, orthodontic/pediatric dentistry, restorative dentistry, oral and maxillofacial surgery, endodontics, dental hygiene, periodontics, and special care dentistry [13]. Not only dental instruments but also teeth may be aspirated: spontaneous molar aspiration with cardiac arrest (before resuscitation) has been described in a patient with poor dentition [14].

Differential diagnosis between inhalation and ingestion may be difficult when no cough or suffocation occurs. In this context, we report the case of a tooth aspiration by a four-year-old boy with Down syndrome, which caused a severe chest infection. Practical recommendations to prevent such incidents and their complications are reviewed.

## 2. Case Report

A 4-year-old boy with trisomy 21 came to the Dental Hospital-University service with his parents, following a fall at the family home four days before. The child did not show any loss of consciousness after the trauma and was up to date with his vaccines. He had already seen a general practitioner and was referred in emergency to a specialist for oral care. His parents described bleeding and crying when the fall occurred and serious difficulties with eating since it happened. The clinical examination was complicated by the poor cooperation of the child. No lesion of the exobuccal integuments was found, but an enamel fracture of the 51 (upper right incisor) and a complex coronal fracture of the 61 (upper left incisor) with pulp exposure and intrusion were noticed. The swollen aspect of the vestibular gingiva with a necrotic appearance led to the decision to extract the 61 under local anesthesia and nitrous oxide sedation. It had not been possible to obtain a correct X-ray. Despite the use of equimolar oxygen nitrous oxide mix, the patient was very restless. Due to a sudden head movement by the child just after the extraction, the tooth slipped out of the forceps, fell on the tongue, and disappeared into the pharynx. The child performed a swallowing movement without any sign of choking or coughing and thereafter presented normal postoperative behavior while playing in the waiting room. As no obvious signs of respiratory distress were shown by the young boy, it was thought that he had swallowed the tooth. He was discharged with supervisory advice.

**Figure 1:**
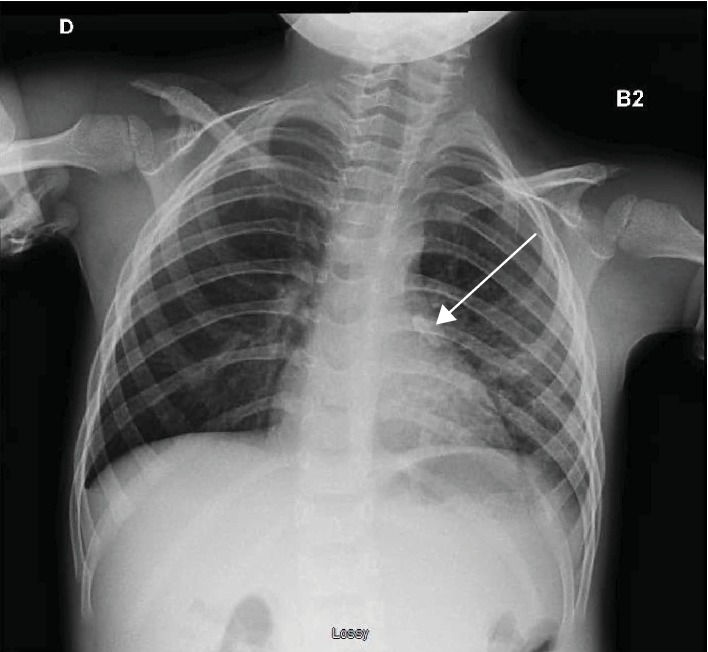
The lung X-ray performed three weeks after tooth extraction shows the tooth inside the left lung.

Three weeks later, the patient was admitted to the Emergency Department because of a regular cough and fever that had lasted for several days despite three different antibiotic therapies. The cardiopulmonary auscultation was normal. A chest radiograph confirmed the presence of a foreign body inside the left lung (Figure 1). It had caused a lung infection of the left lower pulmonary lobe, which was treated by antibiotics (clavulanic acid and penicillin) and anti-inflammatories (prednisolone), before surgery. Two endoscopies under general anesthesia were necessary to recover the tooth. The first one failed due to the presence of purulent secretions and a bleeding granuloma in contact with the tooth that made it difficult to remove. The parents had been informed that, in case of a second failure, more invasive surgery with thoracotomy would be necessary. After the success of the second endoscopy, the child recovered fully. One-year follow-up showed there was no longer any sign of lung infection. This adverse event was discussed at the morbidity and mortality conference of the Dental Hospital service.

## 3. Discussion

Reports about inhalation of foreign bodies and focusing attention on Down syndrome children [15, 16] and neurologically impaired children [17] remain very rare. However, these patients are at a higher risk because of their pharyngeal and orofacial hypotonia, which can lead to impaired pharynx contraction and coordination, with discoordinated swallow [18, 19]. Moreover, they may exhibit a decreased gag reflex or other impairments in airway protective mechanisms. Diagnosis can be more difficult because they may be less likely to complain about their symptoms or the symptoms may be of a nonspecific nature [17]. Cases of dental (or any other foreign body) aspiration reported in the literature indicate different symptoms, such as nonproductive cough [20], dyspnea [3], wheezing, hemoptysis, cyanosis, or a total absence of symptoms [21, 22]. Ninety percent of obstructions following tooth aspiration are situated in the distal airway and 10% in the upper airway [14], which means that the risk of development of fibrosis encapsulation and infections is high. Pneumomediastinum and subcutaneous emphysema may also occur, as described in an asthmatic young child after aspiration of a pumpkin seed [23]. Finally, Down syndrome patients are prone to infections because of their immune deficiencies, which could explain the severe pneumopathy developed by our patient [24].

The major risk is usually immediate, with asphyxia, suffocation, and choking cardiac arrest, but sometimes, FBA may remain undiagnosed if asymptomatic (in 8% of cases according to Mohammad et al. [5]). Delayed diagnosis can lead to recurrent atelectasis, chest infection, emphysema, or misdiagnosis of asthma [15]. The therapy of choice in cases of presence of a foreign body in upper airways is its retrieval by rigid bronchoscopy, sometimes endoscope assisted for better visualization and manipulation of the foreign body [25]. This procedure always requires general anesthesia and a subsequent hospitalization, especially for young patients. However, the procedure is not harmless, and unfortunately, in some cases, even after successful retrieval, death can occur during the recovery [11].

Teeth are radiopaque and should be easily seen on lung X-rays. Nevertheless, it is still helpful for the physician when parents can recall the event and give information about the foreign body. In our case, as the dental practitioner and the parents thought that the tooth had been swallowed, the foreign body was not immediately identified.

Therefore, the prescription of a lung X-ray should become systematic when a tooth disappears, in cases of suspicion of swallowing or even in the absence of symptoms [20]. The guidelines of the International Association of Dental Traumatology recommend referring the child to a pediatrician in order to exclude the possibility of aspiration if a traumatic avulsed tooth has not been found. Even if we consider minimizing the risk of radiation to the child, following the principle ALDAIP (As Low as Diagnostically Acceptable being Indication oriented and Patient specific), complications of these inhalation incidents justify the need for radiographic exposure.

The recommendations to be published could be the following:

1. Once the aspiration is confirmed, the patient should firstly be leant forward to improve breathing. It is essential that the practitioner and his staff remain calm. The patient must be reassured and carefully evaluated. If symptoms of respiratory distress appear, first aid must be initiated (try to remove the foreign body, perform the Heimlich manoeuver, oxygen supply) and prompt contact with the emergency services must be established
2. Secondly, both clinical and radiological evaluations are required. Urgent management with a flexible or hard fiber optic bronchoscope should be performed to remove the object that can obstruct the airway or cause lung infection
3. Thorough documentation of the accident is required. Further documentation may include notation of all medical care, copies of radiographic reports confirming the diagnosis, and removal reports for the objects. The patient's whole medical and dental history should be reviewed
4. Finally, in cases of tooth extraction, prevention should include appropriate anesthesia and sedation, proper body and head positioning, adequate lighting, and four-handed dentistry with an attentive assistant helping to maintain the head if necessary. Sterile gauze or appliances like Isovac® (Innerlite Inc.) may be used as a protective barrier in the oral cavity, distal to the working area

Conscious sedation with nitrous oxide preserves the protective reflex of airways, but the backward inclined position of the patient promotes the fall of objects directly into the posterior part of the oral cavity, triggering a swallowing reflex or inhalation instead of deglutition [13]. The indication of removing a tooth in an uncooperative, handicapped child with insufficient sedation may be questioned. It would be less risky to delay the extraction and use a deeper sedation. However, in an emergency situation, decision-making must be considered in the difficult context of pain, child complaints, and feeding difficulties.

## 4. Conclusion

Foreign body aspiration is a common complication that should be anticipated, especially for special care in dentistry. At the slightest doubt after an object has disappeared, a chest X-ray must be prescribed immediately. This case of tooth inhalation confirms the importance of a multidisciplinary organization inside the hospitals, to take care of patients with handicap and manage the care complications as well as possible.
